# Cell Culture-Based Assessment of Toxicity and Therapeutics of Phytochemical Antioxidants

**DOI:** 10.3390/molecules27031087

**Published:** 2022-02-06

**Authors:** Peace C. Asuzu, Nicholas S. Trompeter, Carlton R. Cooper, Samuel A. Besong, Alberta N. A. Aryee

**Affiliations:** 1 Department of Human Ecology (Food Science and Biotechnology Program), College of Agriculture, Science and Technology, Delaware State University, Dover, DE 19901, USA; pcasuzu17@students.desu.edu (P.C.A.); sbesong@desu.edu (S.A.B.); 2 Department of Biomedical Engineering, University of Delaware, Newark, DE 19716, USA; ntromp@udel.edu; 3 Center for Translational Cancer Research, Department of Biological Sciences, University of Delaware, Newark, DE 19716, USA; crcooper@udel.edu

**Keywords:** medicinal plants, antioxidant activity, cell lines, cytotoxicity, apoptosis, bioprinting

## Abstract

Plant-derived natural products are significant resources for drug discovery and development including appreciable potentials in preventing and managing oxidative stress, making them promising candidates in cancer and other disease therapeutics. Their effects have been linked to phytochemicals such as phenolic compounds and their antioxidant activities. The abundance and complexity of these bio-constituents highlight the need for well-defined in vitro characterization and quantification of the plant extracts/preparations that can translate to in vivo effects and hopefully to clinical use. This review article seeks to provide relevant information about the applicability of cell-based assays in assessing anti-cytotoxicity of phytochemicals considering several traditional and current methods.

## 1. Introduction

Cancer is one of the leading causes of death worldwide. It is the first or second leading cause of death prior to age 70 in 112 of 183 countries and third or fourth leading cause in a further 23 countries, according to World Health Organization (WHO) estimates in 2019 [1]. The rising prominence of cancer as a leading cause of death in combination with limited clinical interventions clearly compromises the effects of treatment on population trends in cancer mortality, even in developed countries [2]. Although a combination of screening and treatment is progressively effective in reducing mortality from some cancers, an expected global cancer burden of 28.4 million cases in 2040, a rise of 47% from 2020 values, necessitates the development of new tools to address the unmet needs in cancer management [1]. Although newer, more specific treatments are showing promising results, they can be expensive, and further research is required to determine how to best use these drugs, as well as the toxicities associated with their use [3].

The most common types of cancer treatments available today are chemotherapy, surgery, and radiotherapy. Chemotherapy is curative in subsets of patients presenting with advanced disease, including Hodgkin’s and non-Hodgkin’s lymphoma, acute lymphoblastic and acute myelogenous leukemia, germ cell cancer, small cell lung cancer, ovarian cancer, and choriocarcinoma [4,5]. Chemotherapy has also been used as a neoadjuvant therapy to reduce the size of solid tumors before surgical removal, and adjuvant therapy has been used after surgery or radiotherapy, with promising results [4]. However, for some other advanced cancers, including prostate cancer, a curative treatment regimen is yet to be discovered. Scientists are returning to the drawing board to find new therapies or new combinations of therapies to further improve cancer treatment outcomes.

Increased reactive oxygen species (ROS) levels have been found in almost all cancers and are thought to play an important role in the initiation and progression of cancers [6]. These highly reactive ions and molecules are produced during normal metabolism of cells but are present in higher levels in cancer cells due to increased metabolic activity, mitochondrial dysfunction, peroxisome activity, increased cellular receptor signaling, oncogene activity, increased activity of oxidases, cyclooxygenases, lipoxygenases and thymidine phosphorylase, or through crosstalk with infiltrating immune cells [6]. ROS are managed under normal physiological conditions, through detoxification by non-enzymatic molecules such as glutathione, or through antioxidant enzymes, which specifically scavenge different kinds of ROS [6]. With increasing interest in natural products, scientists continue to consider plants, which are natural sources of exogenous antioxidants, as possible sources of effective treatments for different cancers.

## 2. Medicinal Plants in Cancer Treatment and Management

Phytochemicals are classified as primary or secondary metabolites based on their role in plant metabolism [7]. Secondary metabolites are chemically active compounds including alkaloids, anthocyanins, flavonoids, terpenoids, tannins, steroids, saponins, coumarins, phenolics and antioxidants. These are often produced in response to stress, are more complex in structure, and are less widely distributed than the primary metabolites [7,8]. They are pharmacologically active as anti-oxidative, anti-allergic, anti-bacterial, anti-fungal, anti-diabetic, anti-inflammatory and anti-carcinogenic compounds [8,9,10]. It is common for a single plant to produce many secondary metabolites with a wide range of chemical and biological properties, providing a range for bioactive substances [10].

In the last decades, several plants have been confirmed to contain chemo-preventive and therapeutic agents for various cancers [11,12,13,14,15,16,17,18,19,20]. These studies show the effectiveness and synergistic effects of phytochemicals in plant extracts in various diseases [15,21,22]. Researchers have discovered that polyphenols are good antioxidants, capable of neutralizing the destructive reactivity of reactive oxygen/nitrogen species produced as byproducts of metabolism [23]. In addition, epidemiological studies have revealed that polyphenols provide significant protection against development of several chronic conditions such as cardiovascular diseases (CVDs), cancer, diabetes, infections, aging and asthma [23]. Phenolic phytochemicals are the largest category of phytochemicals and the most widely distributed in the plant kingdom [24].

There have been studies to examine the effect of crude plant extracts or fractions containing phenolic compounds on cancer cells to test the hypothesis that potent antioxidants possess anticancer potential. Some of these studies revealed that the efficacy of phenolic compounds in inhibiting cancer activity differs based on the structure of the phenolic compound and its molecular target [25]. Phenolic compounds can directly scavenge free radicals after entering cells and activate several cellular signaling pathways (CSP), including nuclear factor erythroid-2 (NFE2)-related factor 2 (Nrf2)-Kelch-like ECH associated protein 1 (Keap1) complex [26]. When activated, the Nrf2-Keap1 complex induces cellular defense mechanisms, including phase II detoxifying enzymes, phase III transporters, anti-oxidative stress proteins, and other stress-defense molecules that protect normal cells from ROS and reactive metabolites of carcinogenic species [26]. Another CSP that can be activated by phenolics is the mitogen-activated protein kinases (MAPKs) cascade, which helps regulate proliferation, differentiation, stress reduction, and apoptosis in cells [26].

Anticancer activity of phenolic compounds has been studied with the use of crude extracts containing mixtures of phenolic compounds and with isolated phenolic compounds. Some examples of crude extracts with reported anticancer activity include: *Pandanus amaryllifolius* extracts containing gallic acid, cinnamic acid and ferulic acid, with reported in vitro inhibition of breast cancer cell lines [25]. Several *Teucrium* species extracts containing hydroxycinnamic acid derivatives, phenylethanoid glycosides, flavonoid glycosides, and flavonoid aglycones, with reported antiproliferative and proapoptotic activities in HCT-116 colon cancer cell lines [27,28]; *Baccharis trimera* extracts containing gallic acid, pyrogallol, syringic acid and caffeic acid, with reported suppression of tumor cell colony formation and proliferation of SiHa cell line (isolated from a primary uterine squamous cell carcinoma); and Prunus africanus extracts, containing artraric and ferulic acids and N-butylbenzene-sulfonamide (NBBS) with reported antiproliferative effect on prostate cancer cells [15,29]. 

Over 60% of currently used anti-cancer agents are estimated to be derived from natural sources, such as plants, marine organisms, and microorganisms [15,17,30,31]. Good examples of plant sources include *Prunus africana* [15,17], African cherry (*Prunus africana* (Hook.f.) Kalkman) or *Pygeum africanum* (Hook. f.), bitter almond, African prune, and red. Besides its use for timber, it is employed as a medicinal plant, whose leaves, roots and bark are used in traditional medicine in Africa [15,32,33,34,35]. This is not surprising since various bioactive substances with anti-inflammatory, anti-cancer, and anti-viral properties have been identified in different members of the genus *Prunus* [33,35,36,37]. Many phytochemicals from medicinal plants have been discovered to have significant anticancer properties, and many more are yet to be discovered.

## 3. Determining Anti-Cancer Potential of Phytochemicals

Phytochemicals and their derivate metabolites present in plants have been shown to possess several beneficial effects in humans. Some of the more widely known phytochemicals with anticancer properties include vincristine, vinblastine, camptothecin, bleomycin, paclitaxel, and Taxol among others [30,38]. Different mechanisms have been proposed for the anticancer effect of various phytochemicals, with some exerting additive and/or synergistic effects with other phytochemicals. Some of the mechanisms that have been identified include selective killing of rapidly dividing cells, targeting of atypically expressed molecular factors, anti-oxidation, modification of cell growth factors, and inhibition of inappropriate angiogenesis and induction of apoptosis [38]. An example is ellagic acid, found in pomegranates, which induces apoptosis in prostate and breast cancer cells and suppresses metastatic processes of many cancer types [38]. Curcumin, found in turmeric, is attributed to cause apoptosis in cancer cells without cytotoxic effect on healthy cells via several mechanisms, including the regulation of cell proliferation, cell survival, and caspase activation pathways [26,39]. While many phytochemicals that can serve as anticancer drugs by themselves are yet to be discovered, those already discovered can serve as models for the preparation of more effective formulations by applying methods such as total or combinatorial synthesis, or biosynthetic pathway manipulation [16,30]. This concept was applied to overcome the severe toxicity of earlier formulations of paclitaxel by utilizing an albumin-bound nanoparticle technology, which concentrates the drug in tumors [16]. With new and more effective technologies and better understanding of cancer biology, phytochemicals and their derivatives are bound to play a pivotal role in cancer chemotherapy.

### 3.1. Cancer Cell Lines

Cancer cell lines are useful tools because they provide a multifaceted model of the biological mechanisms involved in cancer development and progression. The use of cancer cell lines improved the knowledge of deregulated genes and signaling pathways involved in cancer progression; cell lines have also been used to define potential molecular markers for cancer screening and prognosis [40]. Numerous cell lines with their unique properties and characteristics are currently available for in vitro study of different types of cancer [41].

Cell lines are easy to handle and manipulate genetically/epigenetically by using demethylation agents, small interfering ribonucleic acid (siRNA), expression vectors, and they can be pharmacologically manipulated through cytostatics (cell growth inhibitors). Cell lines are homogenous, providing identical tumor cells for easier analysis unlike in heterogeneous solid tumors. However, to imitate in vivo tumor characteristics as closely as possible, a cancer cell line panel representative of the heterogeneity observed in the primary tumors can be used. Cancer cell lines are pure populations of tumor cells and have a high degree of similarity with the initial tumor. Because of the homogeneity of cell lines, results of experiments using correct conditions are easily reproducible [40]. In addition, there is a substantial number and variety of cancer cell lines available (Table 1). Despite these advantages, some drawbacks of using cancer cell lines include cross-contamination with HeLa cells, genomic instability leading to differences between the original tumor and the specific cell line, changes in the morphology, gene expression, and cellular pathways of cell lines from culture conditions required to maintain them (i.e., culture adaption), and infections with mycoplasma [40]. Furthermore, it is difficult to establish long-term cancer cell lines of certain types of tumors, including prostate cancer tumors [40,42]. The limited number of cell line models for prostate cancer research stems from the difficulty in propagating prostate cancer cells in vitro for extended periods. Investigators have been able to generate only seven cell lines that were previously available through public cell line repositories, but these do not represent the spectrum of clinical disease. New cell lines, which demonstrate the commonly observed clinical phenotypes, are clearly needed [42].

Since the isolation of the first cell line in the 1950s (i.e., HeLa cells), a variety of cancer cell lines have been developed for preliminary drug testing [43]. The different cell lines require different media, growth factors, and supplements to remain viable over time, as the constituents of culture media affect the cell lines. In a study by Kim et al., human breast cancer cells (MDA-MB-231) cultured in minimum essential medium (MEM), Dulbecco’s modified Eagle’s medium (DMEM), or Roswell Park Memorial Institute (RPMI)-1640 medium and containing different concentrations of fetal bovine serum (FBS) or different sera (equine or bovine) showed significant changes in gene expression [44]. They reported that about 25% of genes were expressed at significantly different levels by cells grown in MEM, DMEM, or RPMI-1640 media based on genome-wide expression analysis [44]. In another study, lung cancer cells (A549) and hepatocellular cancer cells (HepG2) cultured in Ham’s F-12 nutrient mix (F12), RPMI, DMEM, and MEM revealed a significantly increased proliferation rate for A549 cells in DMEM compared to the other media tested, and the lowest rate for both A549 and HepG2 cells in MEM, confirmed by assaying conditioned media for basal level ATP at 72 h [45]. This underscores the significant effect of growth conditions and/or environment on cells in drug discovery experiments, and the need for specificity to ensure results are reproducible.

There are a handful of prostate cancer cell lines in use today, most of which have been established from metastatic deposits [46]. The LNCaP cell line, isolated from a subclavian lymph node metastasis of prostate cancer, maintains several key markers including prostate-specific antigen (PSA), prostate specific membrane antigen (PSMA) and the androgen receptor (AR) [47]. The LNCaP cell line is androgen sensitive (AS) and expresses AR and PSA mRNA/protein [41,48]. It has a doubling time of 60–72 h, is responsive to TGF-α, EGF and IGF-1, which are known to promote cancer development and progression, and has a 50% success rate after xenografting, with a tumor doubling time of 86 h when combined with a Matrigel™ formulation [41]. In another study, LNCaP cells, among others, were injected subcutaneously between the scapulae of pfp^−/−^/rag2^−/−^ double knock-out mice, resulting in primary tumor growth and pulmonary metastases in 100% of LNCaP-injected mice, and detection of DNA of 266 circulating tumor cells (CTC) per mL of blood and 35 disseminated tumor cells (DTC) per mL bone marrow after Alu-PCR analysis [49]. Through passage and hormonal manipulation in vivo, the lineage-related LNCaP sublines have resulted in a series of cells that mimic the progression of prostate cancer from the original AS LNCaP cell line to the androgen-independent (AI) C4-2 and C4-2B cell lines [47,50]. 

An AI cell line, C4-2, reproducibly and consistently follows the metastatic patterns of hormone-refractory prostate cancer by producing lymph node and bone metastases when injected either subcutaneously or orthotopically in either hormonally intact or castrated hosts. This model enables the study of factors that determine the predilection of prostate cancer cells for the skeletal microenvironment [50]. These C4-2 cells have a doubling time of about 48 h, are androgen independent, express an androgen receptor, metastasize to lymph nodes, and produce PSA [41,46]. The AI C4-2 cell line differs from its parent AS LNCaP, with differential expression of 38 genes between the two cell lines (≥2-fold change, 95% CI), 14 of which expressed at higher levels in LNCaP than in C4-2 cells, while the remaining 24 were expressed at lower levels in LNCaP than in C4-2 cells. In addition, the AI C4-2 cell line is highly tumorigenic and metastatic, including spontaneous metastasis to bone, whereas the AS LNCaP cell line is only weakly tumorigenic and is non-metastatic [47]. 

**Table 1:** Some cell lines used in determining anticancer potential of selected medicinal plants.

Cell Line	Culture Medium	Supplementation	Medicinal Plant	Reference
Androgen-dependent growing human prostate cancer cell line, LNCaP (lymph node prostate cancer)	Roswell Park Memorial Institute (RPMI) 1640 medium	10% fetal calf serum (FCS), penicillin (100 U/mL), streptomycin (100 U/mL), and 25 mM 4-(2-hydroxyethyl)-1-piperazineethanesulfonic acid (HEPES)	*Prunus africana*	[51]
Human hepatoma Hep3B cells stablyexpressing green fluorescent protein (GFP)-AR or yellow fluorescent protein (YFP)-AR-cyan fluorescent protein (CFP)	Minimum Essential Medium Eagle, alpha modification (ɑ-MEM)	5% FCS, 2 mM L-glutamine, penicillin (100 U/mL), streptomycin (100 U/mL), and Genticin (G418)	*Prunus africana*	[51]
Monkey kidney CV1 cells	Dulbecco’s modified Eagle’s medium (DMEM)	10% (*v*/*v*) FCS, penicillin (100 IU/mL) and streptomycin (100 IU/mL)	*Prunus africana*	[37]
Mouse mammary breast cancer cell line, mouse colon cancer cell line and Vero cells (monkey kidney cells)	Earl’s Minimum Essential Media (EMEM)	Penicillin, streptomycin and 10% fetal bovine serum (FBS)	*Prunus africana*	[52]
Human embryonic kidney cells, HEK293	EMEM	Glutamine, 10% FBS and antibiotics (100 μg/mL penicillin, 100 μg/mL streptomycin)	*Moringa oleifera, Prunus africana*	[53]
Colorectal adenocarcinoma cell line, Caco-2	EMEM	Glutamine, 10% FBS and antibiotics	*Moringa oleifera, Prunus africana*	[53]
Hepatocellular carcinoma cell line, HepG2	EMEM	Glutamine, 10% FBS and antibiotics	*Moringa oleifera, Prunus africana*	[53]
HepG-2, Caco-2 and the non-cancer cell line HEK293	EMEM + glutamine	10% FBS, 100 μg/mL penicillin and 100 μg/mL streptomycin	*Prunus africana*	[54]
Human prostate carcinoma LNCaP cells	RPMI-1640 medium	10% (*v*/*v*) FCS, 1% (*v*/*v*) penicillin and streptomycin, 1% (*v*/*v*) L-glutamin and 1% (*v*/*v*) sodium pyruvate	*Prunus africana*	[55]
Human prostate carcinoma cell lines PC3, PC3-ARwt	DMEM	10% (*v*/*v*) FCS, 1% (*v*/*v*) penicillin and streptomycin, 1% (*v*/*v*) L-glutamin (and 600 μg/mL geneticin for PC3- ARwt)	*Prunus africana*	[55]
Human prostate cancer C4-2 cells	DMEM	10% (*v*/*v*) FCS, 20% F12, 5 μg/mL Insulin, 13.6 pg/mL T3 (3,3′,5-triiodo-L-thyronine sodium salt), 5 μg/mL apotransferrin, 0.25 μg/mL Biotin, 1% (*v*/*v*) penicillin and streptomycin	*Prunus africana*	[55]
Human prostatic myofibroblasts and fibroblasts (HPMF)	Endothelial basal medium MCDB 131	1 × L-glutamine, 5% FCS, 1 × MEM vitamins solution, 1× insulin-tranferrin-selenium liquid media supplement, and 1% (*v*/*v*) antimycotic/ antibiotic solution	*Prunus africana*	[56]
Madin-Darby canine kidney epithelial cell line (MDCK cells)	DMEM	1 × L-glutamine, 5% FCS and 1% (*v*/*v*) antibiotic solution	*Prunus africana*	[56]
Vero E6, CT 26-CL 25 colon cancer cells and Hep2 throat cancer cells	MEM medium	10% FBS, 1% L-glutamine and 1% antibiotic solution	*Prunus africana*	[57]
Human ileoceacal adenocarcinoma, HCT-8 cell line	RPMI-1640	10% heat inactivated FBS, 2 mM L-glutamine, 50 μg/mL of penicillin-G, and 50 μg/mL of streptomycin sulfate	*Moringa oleifera*	[18]
Human breast cancer, MDA-MB-231 cell line	DMEM	10% heat inactivated FBS, 2 mM L-glutamine, 50 μg/mL of penicillin-G, and 50 μg/mL of streptomycin sulfate	*Moringa oleifera*	[18]
Human B-lymphoblastoid cells, Raji	RPMI-1640	10% fetal calf serum (FCS) containing n-butyric acid (3 mM) and teleocidin B-4 (50 nM)	*Moringa oleifera*	[58]

### 3.2. Recent Advances in Cell Culture Models for Testing Anticancer Drugs

**Figure 1:**
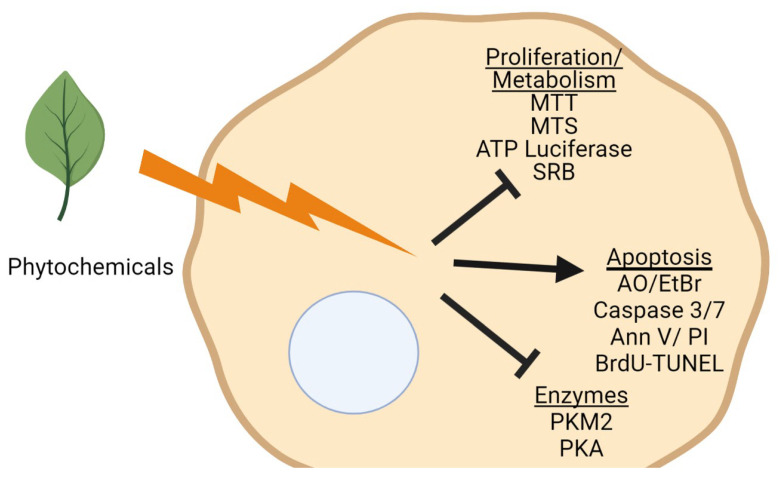
Anti-cancer bioassays to assess in vitro cellular effects of phytochemicals. The inherent antioxidant activities of phytochemicals may elicit anti-tumorigenic effects through either the inhibition of proliferation/cell metabolism, induction of cell apoptosis, or the inhibition of cancer associated enzymes. Assays to assess each cellular function are listed below each underlined function. MTT: 3(4, 5-dimethylthiazol-2-yl)-2, 5-diphenyltetrazolium bromide; MTS: 3-(4, 5-dimethylthiazol-2-yl)-5-(3-carboxymethoxyphenyl)-2-(4-sulfophenyl)-2H-tetrazolium; SRB: sulforhodamine B; AO/EtBr: Acridine orange/ethidium bromide; Ann V/PI: Annexin V/Propidium Iodide; BrdU-TUNEL: bromodeoxyuridine-Terminal deoxynucleotidyl transferase-mediated d-UTP nick end labeling; PKM2: pyruvate kinase M2; PKA: protein kinase A. (Created with BioRender.com.)

In vitro anti-cancer screening has long been used by researchers as a rapid tool in screening natural and synthetic compounds for drug development [53]. To assess preliminary anti-cancer activity in terms of cell viability, the 3(4, 5-dimethylthiazol-2-yl)-2, 5-diphenyltetrazolium bromide (MTT) and 3-(4,5-dimethylthiazol-2-yl)-5-(3-carboxymethoxyphenyl)-2-(4-sulfophenyl)-2H-tetrazolium (MTS) in vitro cytotoxicity assays are considered two of the most economic, reliable, and convenient methods (Figure 1) [53,59]. This is based on their ease of use, accuracy, rapid indication of toxicity, and sensitivity and specificity [59]. Both assays are in vitro whole cell toxicity assays that employ colorimetric methods for determining the number of viable cells based on mitochondrial dehydrogenase activity measurement and differ only in the reagent employed [59]. 

In the MTT assay, the MTT salt is bio-reduced by dehydrogenase inside living cells, using the succinate-tetrazolium reductase system, to form a colored formazan dye, while a similar bioconversion using the MTS salt and phenazine ethosulfate as an electron coupling reagent occurs in the MTS assay [53,59]. In addition, the MTT assay requires the addition of solubilizing agents to dissolve the insoluble formazan product, while the MTS assay generates a water-soluble formazan product. The quantity of the colored product is directly proportional to the number of live cells in the culture since only metabolically active cells can reduce the MTT/MTS reagent to formazan [53,59]. The MTT and MTS assays assess the toxicity of a compound to cells but not anti-cancer activity. In addition, the MTT reagent is cytotoxic and subject to interference by chemical compounds such as vitamin A and C, which can lead to an under- or overestimation of cell viability, respectively [60,61]. MTS can be chemically reduced by reducing agents such as gallic acid, and the absorbance measured in the MTS assay is influenced by the incubation time (ideally 1–3 h), cell type, and the proportion of MTS reagent to cells in culture, hence the cell number [60,62]. Therefore, these factors must be considered in interpreting the results from these tests.

The sulforhodamine B (SRB) assay is a rapid, sensitive, and inexpensive method for determining cell growth, utilizing a bright pink anionic dye that binds electrostatically to basic amino acids of trichloroacetic acid fixed cells. The protein-bound dye is extracted with Tris (tris (hydroxymethyl) aminomethane) base to quantify the protein content indirectly with spectrophotometry [63]. The endpoint of the SRB assay is non-destructive, stable, does not require time-sensitive measurement, and it is comparable with other fluorescence assays [60,64]. However, it is labor intensive, requiring several washing steps [63,65].

A known characteristic of cancer cell growth and metastasis is the ability of the cells to escape apoptosis because of a mutation in tumor suppressor genes. Induction of apoptosis is thus used as an important indicator of the ability of chemotherapeutic agents to inhibit tumor growth and progression. The acridine orange/ethidium bromide (AO/EB) apoptosis assay is used to study changes in cellular and nuclear morphology and characteristics of apoptosis under a fluorescent microscope [53]. Both AO and EB bind to DNA and RNA by intercalation between adjacent base pairs, but AO stains both live and dead cells while EB stains dead cells only. Live cells appear green under the microscope, and early apoptotic cells have a bright green nucleus due to chromatin condensation and nuclear fragmentation, while late apoptotic cells appear orange because they take up EB, and necrotic cells will stain orange but have a normal nuclear morphology [53,66]. After cells are counted under the microscope, an apoptotic index is calculated.

The living status of a cell can be determined by measuring the amount of ATP in the cell, since ATP is necessary for life and function of all cells, and levels of cytoplasmic ATP decrease in cases of injury and hypoxia. After a cell is lysed, ATP is free to react with luciferin and luciferase, producing high quantum chemiluminescence that is linearly proportional to the ATP concentration under optimum conditions. Compared to the MTT assay, the luciferase assay had higher sensitivity and reproducibility over several days and was able to detect the viability of cells with cell counts as low as 2000 cells/well compared to a minimum of 25,000 cells/well required for the MTT assay mentioned above. The ATP assay has been reported as sensitive compared to MTT and calcein assays, used to determine the potency of cytotoxic agents [67]. This high sensitivity of the ATP assay allowed for detection of cytotoxic agent-induced ATP breakdown after incubation periods as short as 1 h, which provides an additional advantage over the MTT assay that requires approximately 72 h of incubation. A further advantage of the ATP assay was the short measurement time of 15 s per well, compared to the MTT assay, which required a 1–2 h solubilization step of the formazan before an absorption measurement [67]. However, the ATP assay cannot differentiate between cytostatic and cytotoxic cellular effects [64].

Many anticancer drugs in use today inactivate target cells by inducing apoptosis [68]. As one of the later steps in apoptosis, DNA fragmentation, a process resulting from the activation of nucleases that break down DNA into small fragments, can be used as a measure of anticancer agent bioactivity [68]. When anticancer agents break down DNA, they expose many 3′-hydroxyl ends to which fluorescein deoxythymidine analog, 5-bromo-2′-deoxyuridine 5′-tri-phosphate (BrdUTP) molecules attach, with the help of terminal deoxynucleotidyl transferase (TdT). One of the TdT dUTP Nick-End Labeling (TUNEL) assay methods involves the attachment of a fluorescein deoxythymidine analog, 5-bromo-2′-deoxyuridine 5′-triphosphate (BrdUTP) molecule [68]. After it is assimilated into the DNA, BrdU can be detected by an anti-BrdU antibody using standard immunohistochemical techniques, fluorescence microscopy or flow cytometry [68]. TUNEL assays have been used in the evaluation of many anticancer compounds, including derivatives of betulinic acid and botulin and 5-fluorouracil [68]. The harsh denaturing conditions necessary for the binding of anti-BrdU cause cell disruption and protein degradation, which is a limitation, particularly if concurrent protein content measurement or molecular analysis is required [64,69]. 

Although two-dimensional (2D) cell culture systems involving the growth of a monolayer of cells on a plastic surface or in vivo animals were the standard for drug testing, data from 2D models are often misleading, resulting in difficulties with translational efficacy in vivo [70,71]. This is mostly because while convenient, 2D systems are over simplistic representations of the in vivo complex tissue architecture, which fail to incorporate the biochemical and biomechanical crosstalk between tumors and the surrounding tumor microenvironment [70,72,73]. The absence of drug transport barriers, extracellular matrix and blood vessels, immune cells, gradients of oxygen tension, extracellular pH, nutrients, catabolites present normally in tumor conditions in vivo, coupled with short-term culture conditions in 2D systems, may select for cytotoxic drugs that prove insufficient in pre-clinical and clinical settings [70,72,74]. Therefore, in a bid to overcome these limitations and avoid the ethical concerns involving animal testing, new test systems such as the Boyden’s chamber, three-dimensional (3D) cultures, microfluidic device systems, and models created using 3D bioprinting were developed [72]. 

One of the new systems used to model the complex in vivo intercellular interactions in vitro is the Boyden chamber, consisting of two chambers containing media and partitioned by a semi-permeable membrane [72,75]. To study cell migration in a Boyden chamber, cells are seeded in the upper chamber and allowed to migrate under the influence of a concentration gradient of chemotactic substances added to the media in the lower chamber media [75,76]. Cell migration is assessed by measuring the optical density of labeled cell extracts and corresponds to the effectiveness of the biologically active substance [75]. The Boyden chamber was used to assess and compare the invasive activity of spheroids containing only tumor cells and spheroids containing a mixture of tumor and stem cells, showing an increased invasion of the heterogeneous spheroids when compared to spheroid containing only tumor cells [72,77]. Although easy to use, the Boyden chamber does not allow for direct cell–cell interactions, limiting its ability to fully reproduce in vivo conditions and creating a preference for other evolving methods such as 3D culture and microfluidic systems [75]. 

An ideal 3D system would mimic a specific solid tumor microenvironment, where cells are able to replicate and interact with other cells, while promoting differentiation [78]. Most 3D systems, however, do not exactly simulate in vivo conditions, although they are more representative than 2D models. Three-dimensional systems may be classified as free-floating anchorage-independent systems, scaffold-based systems, and organoids, which are hybrid 3D culture models composed of spheroids [78]. Regardless of the class of 3D system used, research has shown that cancer cells grown in 3D culture may respond to drug treatment similarly to cancer cells in the native environment [70,72,78]. In addition, differences in apoptotic sensitivity to chemotherapeutic agents have been noted in nonmalignant and malignant mammary cell lines between 2D and 3D cultured cells [79,80]. In another study, BT-549, BT-474, and T-47D breast cancer cells in a 2D culture were less resistant to paclitaxel and doxorubicin compared to a 3D culture of the same cells [72,81]. Three-dimensional cell culture systems also provide an alternative to suspension cultures, which are necessary for growing poorly adherent cancer cells and non-solid tumor cells such as leukemia [70]. In addition, the development of organoid cultures has created more ways to carry out high-throughput drug screening using 3D culture, which may facilitate personalized cancer treatments, biomarker discovery, and mechanistic studies on drug resistance [73]. Organoid cultures have been successfully used to model pancreatic ductal adenocarcinoma from patient derived xenograft tumors and from patient prostate cancer bone metastasis [72].

Bioprinting allows for the creation of various models that mimic the processes that occur in the tumor microenvironment (TME) and is a method for constructing complex 3D biological structures. This is achieved by printing a bio ink composed of an extracellular matrix (ECM) or other synthetic substrate and cells layer-by-layer in a computer-designed pattern [72,82]. In this way, a system that mimics the TME of cervical cancer, triple-negative breast cancer with fibroblasts, and patient-derived cancer cells, fibroblasts, and endothelial cells have been created [82]. Three-dimensional bioprinting has been used to highlight the trophic role of stromal or immune cells in breast cancer cells cultured with fibroblasts in spheroids which remained viable for over 30 d and were resistant to paclitaxel, unlike the homogenous breast cancer spheroids [72]. Three-dimensional bioprinting also makes it possible to study immune cell behavior in the TME. For example, glioblastoma cells in a 3D bioprinting model were shown to polarize actively recruited macrophages in glioblastoma-associated macrophages, enhancing the proliferation and invasiveness of the glioblastoma cells [72]. 

Three-dimensional bioprinting can also be used to design systems that simulate aberrant tumor vascularization to better understand tumor biology and for in vitro drug testing [72,82]. Microfluidic systems involve the use of small devices designed for cell cultures to mimic perfusion, thus allowing for steady supply of oxygen and nutrients to cells and the removal of wastes [72,78]. They make it possible to control fluid flow, temperature, hydrodynamic and hydraulic pressures, shear, and chemical gradients in vitro to simulate physiological conditions in the TME [72]. The device may be designed with a barrier between compartments or a non-physical barrier such as a biomimetic extracellular matrix to divide the compartments in the device [78]. Microfluidic systems can be used to simulate a metastatic model of tumors, allowing researchers to study the effects of anti-metastatic drugs on tumor cell migration. For example, a microfluidic system using collagen–matrigel hydrogel matrices was used to reproduce the microenvironment and experimental conditions to study the migration and invasion of H1299 lung adenocarcinoma cells [72]. Different forms of microfluidic systems such as well plate, droplet, and continuous flow microfluidics are amenable to high-throughput drug screening, making them desirable for anticancer drug screening [83].

### 3.3. Real-Time Assessments of Cell Culture Assays

With the establishment of 3D cultures, there is a need for monitoring and recording the cultures in 3D in real time and over the time needed for progression to occur, namely, in 4D [84]. Real-time image-based analysis of cellular response to drug activity in vitro and in vivo may expedite drug development timelines, decrease costs, provide better understanding of adaptive response and increase clinical predictivity when used with relevant model systems [85]. While traditional time-lapse epifluorescent and confocal microscopes provide detailed temporal and spatial analysis of cellular function, they are restricted to one or a few samples per experiment, limiting their application for drug discovery. However, new generation live-cell imaging microscopes allow for examination of dynamic cellular processes in response to multiple molecular or pharmacological interventions [85]. These include the IncuCyte™; Cell-IQ™ and Biostation CT™, which are equipped with software to remotely control image acquisition, filter optic configurations and image analysis, and are optimized for long-term kinetic studies across multi-well plates [85].

The standard IncuCyte-FLR™ system can accommodate up to six 384-well plates and can automatically monitor cell growth, cell migration into a wounded monolayer, angiogenesis and apoptosis [85]. In a recent study, the Sartorius IncuCyte^®^ system was used to investigate the killing potential of immune cells on cancer cell lines, tracking living cells labeled by a red fluorescent protein, and cell death through the green fluorescent signal generated when apoptotic pathways are activated [86]. The Cell-IQ^®^ system is a fully integrated incubator, with continuous live cell imaging and an automated analysis platform that combines phase-contrast microscopy and fluorescent image acquisition with an analyzer software package for the quantification of migration image data [85,87]. Processes such as cell attachment, migration velocity, migration direction, neurite outgrowth, vesicle formation, angiogenesis and stem cell differentiation have been documented using the Cell-IQ™ system, which allows for a robust kinetic study of phenotypic response to drug treatment [85]. The Nikon Biostation CT™ platform is reported to be the first multi-objective fluorescent and phase contrast microscope combined with automated plate handling robotics within a cell-culture incubator [85]. The Biostation CT is used to demonstrate reduced spheroid migration velocity and suppressed spheroid fusion of human breast cancer cell lines BT474 and T47D when exposed to trastuzumab and paclitaxel, correlated by ATP quantification cell viability testing [79].

The ability of these systems to document real-time drug response in cells have the added advantage of making it easier to quantify transient phenotypic responses, optimize time points for endpoint studies, determine accurate dosing and scheduling regimens, identify cancer cell adaptive responses, and facilitate more robust quantitative analysis from less specimens [85]. The combinations of functions in one place, the ability to maintain steady environmental conditions, and remotely controlled multiple phases of experiments are attractive features of these automated live-cell analysis systems.

## 4. Conclusions

The increasing prevalence of cancers worldwide has made the development of quicker methods to create, develop, and test new anticancer drugs a necessity. Anticancer bioassays have proven to be powerful tools in the drug discovery process and preclinical authentication. However, caution should be exercised in comparing results from the different assays, as they usually target different mechanisms. The choice of an anti-cancer bioassay to use depends, to a large extent, on the researcher’s objective, the target cancer cells and phytochemical composition of the medicinal plant, the availability of reagents and cost of reagents, and the experience of the research team. It is also important that a few known anticancer compounds of known potency are included for comparison with potential medicinal plant extracts, regardless of what method is used for cytotoxicity screening, for objectivity and relevance. Cell-based anti-cancer bioassays have much to offer prior to animal testing. Because of lower cost, the investigator has more control of confronting variables, and models can be developed to predict or approximate the phytochemical (i.e., natural drug) effect in animal and, with additional research, human subjects.

## Data Availability

Not applicable.
